# Urinary nitrate concentration as a marker for kidney transplant rejection

**DOI:** 10.1186/s12882-020-02096-x

**Published:** 2020-10-20

**Authors:** Amy Riddell, John Kirkwood, Miranda Smallwood, Paul Winyard, Beatrice Knight, Lidia Romanczuk, Angela Shore, Mark Gilchrist

**Affiliations:** 1 Institute of Biomedical and Clinical Sciences, University of Exeter, and NIHR Exeter Clinical Research Facility, Exeter, Devon UK; 2 Royal Devon and Exeter NHS Trust, Exeter, UK

**Keywords:** Kidney, Transplant, Nitrate, Biomarker, Rejection

## Abstract

**Background:**

Early identification and treatment of kidney transplant rejection episodes is vital to limit loss of function and prolong the life of the transplanted kidney and recipient. Current practice depends on detecting a creatinine rise. A biomarker to diagnose transplant rejection at an earlier time point than current practice, or to inform earlier decision making to biopsy, could be transformative.

It has previously been shown that urinary nitrate concentration is elevated in renal transplant rejection. Nitrate is a nitric oxide (NO) oxidation product. Transplant rejection upregulates NO synthesis via inducible nitric oxide synthase leading to elevations in urinary nitrate concentration. We have recently validated a urinary nitrate concentration assay which could provide results in a clinically relevant timeframe. Our aim was to determine whether urinary nitrate concentration is a useful tool to predict renal transplant rejection in the context of contemporary clinical practice.

**Methods:**

We conducted a prospective observational study, recruiting renal transplant participants over an 18-month period. We made no alterations to the patients’ clinical care including medications, immunosuppression, diet and frequency of visits. We collected urine samples from every clinical attendance. We assessed the urinary nitrate to creatinine ratio (uNCR) between patient groups: routine attendances, biopsy proven rejection, biopsy proven no rejection and other call backs. uNCR was examined over time for those with biopsy proven transplant rejection. These four groups were compared using an ANOVA test.

**Results:**

A total of 2656 samples were collected. uNCR during biopsy proven rejection, *n* = 15 (median 49 μmol/mmol, IQR 23–61) was not significantly different from that of routine samples, *n* = 164 (median 55 μmol/mmol, IQR 37–82) (*p* = 0.55), or biopsy proven no rejection, *n* = 12 (median 39 μmol/mmol, IQR 21–89) (*P* = 0.77).

Overall uNCR was highly variable with no diagnostic threshold for kidney transplant rejection. Furthermore, within-patient uNCR was highly variable over time, and thus it was not possible to produce individualised patient thresholds to identify rejection. The total taking Tacrolimus was 204 patients, with no statistical difference between the uNCR of all those on Tacrolimus, against those not, *p* = 0.18.

**Conclusion:**

The urinary nitrate to creatinine ratio is not a useful biomarker for renal transplant rejection.

## Background

Kidney transplant rejection remains a problem contributing to graft loss. It is typically asymptomatic making early diagnosis difficult. Transplanted kidneys remain at risk of rejection despite significant advancements in immunosuppresion [1]. Early identification and treatment of rejection episodes is necessary to limit loss of function and prolong the life of the transplanted kidney and recipient [2, 3]. Current practice depends on detecting a creatinine rise. Consequently, graft dysfunction and damage are likely by the time of kidney transplant biopsy and subsequent treatment [3]. A biomarker to diagnose transplant rejection at an earlier time point than current practice, or to inform earlier decision making to biopsy, could be transformative.

In 1996 Smith et al. [4] provided evidence that urinary nitrate concentration is elevated during kidney transplant rejection when compared with urinary tract infection or a non-rejection state. The same group replicated these findings showing that urinary nitrate concentration was elevated up to 5 days before a biopsy diagnosis of rejection was made [5]. It has been demonstrated that urinary nitrate concentration increases as a result of an increased expression of inducible nitric oxide synthase (iNOS) leading to increased production of nitric oxide (NO) in the transplanted organ [6, 7]. This NO is then rapidly metabolised to its stable oxidation product, nitrate. Also, in 1996 Mugge et al. [8] discovered similar findings in their cardiac transplant patients. An increased urinary nitrate concentration was noted with moderate/severe cardiac transplant rejection compared with no rejection, however this only reached statistical significance when a small group of patients who had repeated biopsies were considered separately [8].

Historically the measurement of nitrate in biological fluids has been fraught with problems [9], not least of which is the time taken to generate a result. This likely precluded progression of the work by early pioneers in the field [4, 5, 8]. We have previously validated a spectrophotometric plate method for measurement of nitrate in urine [10] which is capable of accurately determining nitrate concentration in urine in a clinically relevant timeframe for renal transplant rejection.

We sought to determine whether urinary nitrate concentration would be a useful tool to predict renal transplant rejection in the context of current clinical practice by comparing urinary nitrate concentration from routine samples given during stable kidney transplant function with those given at times of kidney transplant rejection.

## Methods

We conducted a prospective observational study collecting urine samples from kidney transplant recipients over an 18-month timeframe.

Potential participants attending the renal transplant clinic at the Royal Devon and Exeter Foundation Trust were identified by Clinicians during routine clinical care. The RDETB (Royal Devon and Exeter Tissue Bank, NIHR Exeter Clinical Research Facility) team were responsible for recruitment and data/sample collection. All participants recruited were provided with a unique study identifier (ID), and all samples and data were stored under this unique ID code for later transfer to the research team.

Renal transplant patients were recruited between April 2017 and September 2018 from the Royal Devon and Exeter Hospital Kidney unit, UK. We recruited consecutive attendees from routine transplant clinics. In addition, we targeted new renal transplant patients who are at the highest risk of rejection as well as targeting those attending for a ‘call back’ visit due to abnormal blood results who had not yet been recruited via the routine clinic.

### Exclusions

- < 18 years old
- Unable to consent

### Inclusion

- Functioning renal transplant

No changes were made to patients’ clinical care by the study team including; medications, immunosuppression, diet or frequency of visits. We recorded the following data: patient age, age and type of transplant, transplant mismatch, immunosuppression, kidney function, original kidney disease, and presence of urinary tract infection (UTI).

### Urine collection

Regardless of their route of recruitment, all participants provided random spot urine samples at every clinical attendance during the study timeframe, whether it be a routine clinic or a ‘call back’. Urine was stored at -80 °C prior to analysis.

- ‘Routine’ samples were those provided from attendance at a pre-arranged clinic appointment.
- ‘Call back’ samples were those received when a patient attended due to being unwell, abnormal blood result or another event.

For analysis the call back group was then further divided into – biopsy proven rejection, Biopsy proven non rejection, and all other call backs (biopsy not performed).

### Urine analysis

The spectrophotometric plate method of measuring urinary nitrate as described by Miranda et al. [10] was used. This method has previously been validated by us in urine against the gold standard ozone based chemiluminescence method [11]. The coefficient of variation of the assay was 8.5% [10, 11]. The lower limit of detection of the assay was 6 μM. For urine samples with an undetectable nitrate concentration, a value of 6 μM was assigned. Creatinine was measured using the Jaffe method in the clinical biochemistry laboratory at the Royal Devon and Exeter Hospital, U.K.

We have divided our patients into 4 groups:

Routine – Consisting of patients who only ever provided ‘routine samples’ during the study time frame. These are the patients who have not yet had a clinical concern for possible rejection and provide data on urinary nitrate before a rise in serum creatinine.

Biopsy proven rejection – Consisting of the patients who had a biopsy which diagnosed rejection.

Biopsy proven no rejection – Consisting of patients who went on to have a biopsy which showed no rejection.

Other (Non-biopsied) Callbacks – These are the patients who were asked back to the hospital for clinical reasons, usually an abnormal creatinine. This is the start of the process which may lead to a biopsy. These patients have been kept in the analysis as they were the group who were of sufficient clinical concern to call them back but who subsequently resolved clinically and did not require a biopsy. If uNCR was to be a useful biomarker of early transplant rejection it would need to be able to discriminate between these call back patients and those which were of such clinical concern that they went on to biopsy. Our analysis of these patients would therefore be informative.

To correct for variation in urinary concentration we calculated the urinary nitrate to creatinine ratio (uNCR). This was done for every routine sample and a mean taken per patient in order that each patient only contributed 1 value to the statistical analysis. The patient mean is used here to then look for deviation from this during cases of biopsy proven rejection. This was then used to calculate the overall median for the routine group. For the biopsy patients only the uNCR on the day of biopsy was used to calculate the overall median for both the groups ‘biopsy proven rejection’ and ‘biopsy proven not rejection’. Each overall median was then compared to the other groups. Statistical analysis was completed using an ANOVA single factor test across all four groups with statistical significance accepted if *p* < 0.05.

We analysed subgroups including patient immunosuppression and presence of UTI. Our validated assay measures both urinary nitrate and urinary nitrite, taken as a whole concentration. Although measuring both, urinary nitrite concentration has been shown to be less than 10% of urinary nitrate concentration in UTI in transplant populations [4].

There is data to suggest that Immunosuppressive medications such as tacrolimus and mycophenolate can affect NOS and therefore urinary nitrate [12, 13], making it relevant to look at this data. Statistical analysis was completed using a T-Test assuming equal variance, with statistical significance accepted if *p* < 0.05.

## Results

Two hundred forty-one participants were recruited and all remained in the study. A total of 2656 samples were collected. From these totals, 77 participants provided 162 samples at call back attendances, with the remainder provided from routine appointments. Twenty-seven separate patients had a biopsy performed, with 15 biopsies confirming rejection. Table 1 shows participant demographics.

**Table 1:** Participant demographics

		Routine (***n*** = 164)	Call Backs (***n*** = 77)		
			Rejection (***n*** = 15)	Non-Rejection (***n*** = 12)	Other call-backs (***n*** = 50)
**Age**	Years	57.5	46	43	52
		20–76	27–63	20–57	20–71
**Sex**	% M	55	40	66	56
**Donor type**	DCD %	27	25	33	26
	DBD %	32	33	8	47
	LKD %	39	46	33	26
**Time since transplant %**	< 1 yr. %	21	60	58	59
	1–5 yr. %	41	13	25	22
	> 5 yr. %	37	26	16	18
Creatinine μmol/l	Mean +/− SD	136 +/− 55	252 +/− 132	238 +/− 110	147 +/− 61

*DCD Donation after Cardiac Death*, DBD* Donation after Brain Death*, LKD* Living Kidney Donation

We found creatinine to be lower in both the Routine and Other Callback groups compared to those biopsied. This is expected from the clinical context, with routine samples being from stable patients, and the other call-backs samples being collected from those with a clinical abnormality detected but who did not continue onto biopsy. The patients in the Other Callbacks Group are therefore likely to include those patients with a lower creatinine rise when compared with those who went on to be biopsied.

**Fig. 1:**
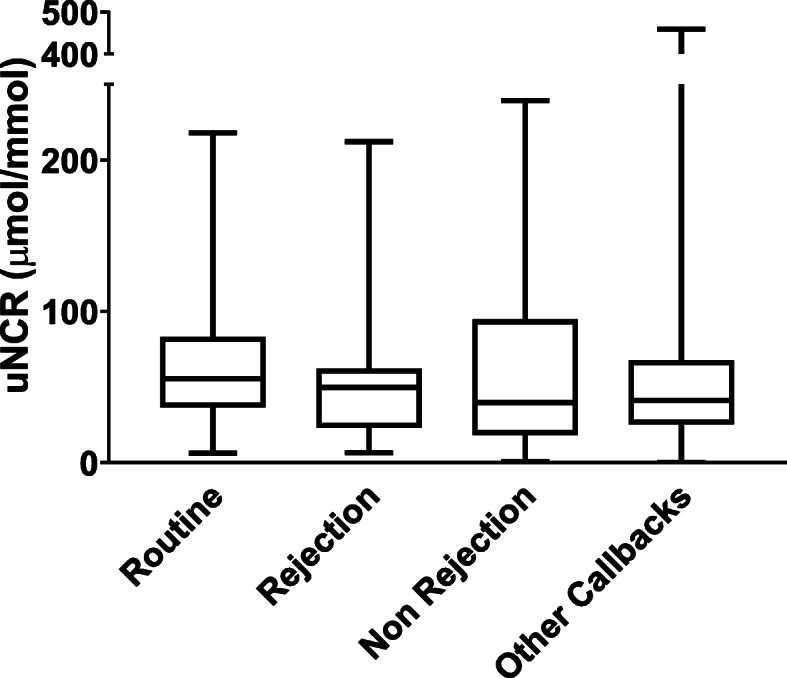
Nitrate: creatinine ratio for each group; Routine, Rejection, Non-Rejection and Other Call-backs. Showing the median, IQR, maximum and minimum values for each patient group. Minimum for ‘Non rejection’ and ‘Other call-backs were 0.73 and 0.26 respectively (μmol/mmol). ANOVA test showed no significant difference between any groups, *p* = 0.98

**Fig. 2:**
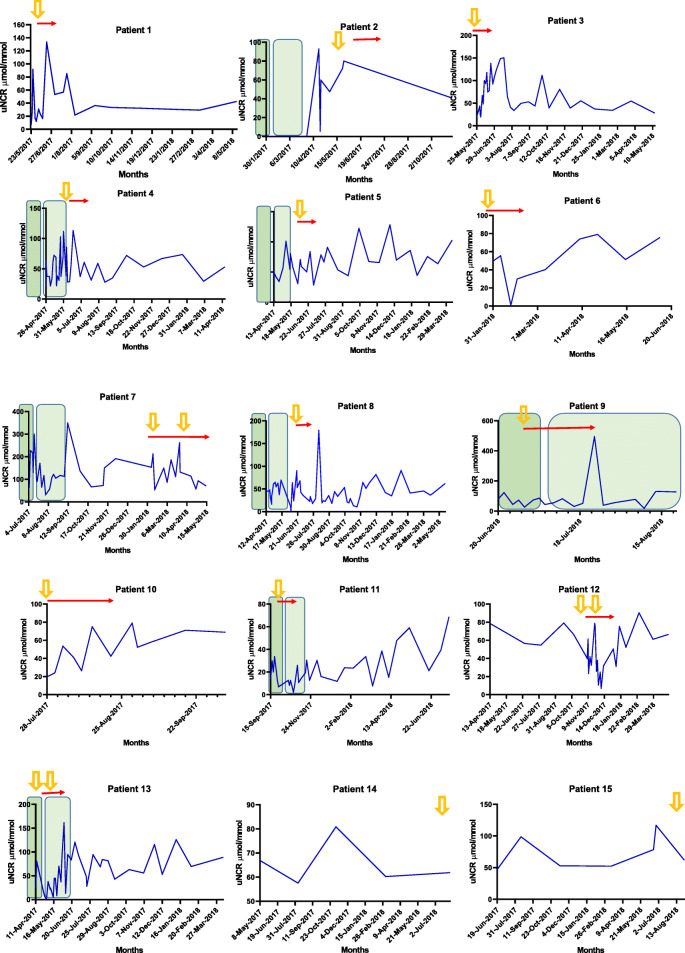
Graph showing the time courses of uNCR values (μmol/mmol) of all samples from 15 biopsy-proven rejection patients.  1st month post transplant  Biopsy proven rejection.  Months 2-3 post tranmsplant  1 month post confirmed rejection

Urinary nitrate data is shown in Table 2. The uNCR did not differ across the four patient groups *p* = 0.98*.* The median uNCR (μmol/mmol) for the Routine Group of patients was 55.44, IQR 37–87, the Biopsy proven rejection Group 49.65, IQR 23–61, the Biopsy proven no rejection Group 39.79, IQR 21–89, and the Other Callbacks Group 41.18, IQR 26–65 (Table 3 and Fig. 1). We analysed the within patient results for all samples provided by those patients with biopsy proven rejection and the uNCR did not appear to be different at the time of biopsy compared with routine samples (Fig. 2 and Table 4).

**Table 2:** Urinary nitrate (μM) concentration according to group

Urinary Nitrate (μmol/l)	Routine (*n* = 164)	Rejection (*n* = 15)	Non-Rejection (*n* = 12)	Other Call-backs (*n* = 50)
Median of patient means	291.14	160.8	202.54	258.81
IQR	250	289	197	273
Min	34	35	6	6
Max	2212	659	598	1144

Number of samples per patient used to calculate the mean (min-max), Routine – 5 (1–33), Rejection 1, Non-rejection 1, Other callbacks 1 (1–5)

**Table 3:** Urinary nitrate to creatinine ratio (μmol/mmol) according to group

uNCR	Routine (*n* = 164)	Rejection (*n* = 15)	Non-Rejection (*n* = 12)	Other Call-backs (*n* = 50)
Median	55.44	49.65	39.79	41.18
IQR	45	38	67	39
Min	5	6	0.7	0.3
Max	217	212	239	459

**Table 4:**
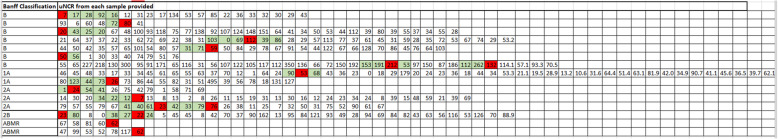
15 Rejection patients; Pattern of uNCR (μmol/mmol) at the time of rejection compared with all the samples they provided.  Date of biopsy proven rejection.  samples within 7 days either side of the date of rejection. Graded as per Banff criteria [3]. *(B=Borderline, ABMR = Antibody mediated rejection)*

In the Other Callback Group only 6 patients had an MSU proven urinary tract infection (UTI). No patients in either of the biopsy groups had an MSU proven UTI. The uNCR results from the time of UTI are not significantly different from those without UTI.

Table 5 shows the total proportion of patients receiving Tacrolimus (*n* = 204) was 83, 93, 91.6 and 88% in groups Routine, Biopsy proven rejection, Biopsy proven no rejection and Other Callbacks respectively. There was no statistical difference between the uNCR of all those on Tacrolimus, using all groups together, against those not, *p* = 0.18 (T test). For those taking Tacrolimus alone, the median uNCR (μmol/mmol) in the Routine Group samples was 53.31, IQR 38.5–81.1, for the Biopsy proven rejection Group 26.69, IQR 19.9–35.1, for the Biopsy proven no rejection Group 59.99, IQR 21.66–168, and for the Other Callback Group 47.01, IQR 26.7–74.99. Similarly, for those taking both Tacrolimus and Mycophenolate the uNCR (μmol/mmol) in the Groups were: Routine samples 56.85, IQR 36.6–89.68, Biopsy proven rejection 49.65, IQR 23.98–87.91, Biopsy proven no rejection 53.43, IQR 39.54–86.93, Other Callbacks 41.66, IQR 24–93.01.

**Table 5:** Results for immunosuppression sub-groups

		Routine	Biopsy proven rejection	Biopsy proven No rejection	Other callbacks
Tacrolimus alone	No.	60	5	4	17
	%	36.58	33.33	33.33	34
	Median	53.31	26.29	59.99	47.01
	IQR	38.4–81.1	19.9–35.1	21.66–168	26.7–74.99
Tacrolimus & Mycophenolate	No.	76	9	6	27
	%	46.34	60	50	54
	Median	56.85	49.65	53.43	41.66
	IQR	36.6–89.6	23.98–87.91	39.54–86.93	24–93.01
Mycophenolate alone	No.	7	1	1	1
	%	4.26	6.66	8.33	2
	Median	62.49	61.84	0.73	30.61
	IQR	27.44–72.63	0	0	0
Neither	No.	21	0	1	3*
	%	12.80	0	8.33	6
	Median	50.77	0	0.11	43.89
	IQR	33.91–60.19	0	0.	40.27–53.39

* = additional 2 patients had unknown data on immunosuppressive medications

## Discussion

This study found urinary nitrate was unable to differentiate renal transplant rejection from stable graft function or other causes of acute graft dysfunction. The uNCR results from the 15 patients who had biopsy proven rejection showed no significant difference from biopsy proven non-rejection or routine groups. The uNCR over time within the same patient showed no significant change during episodes of rejection.

This is likely to be because the biological variability of urinary nitrate is known to be very large [14–16]. The amount of nitrate appearing in the urine is dependent on kidney function [17]. In the context of acute kidney injury, changes in tubular function and glomerular filtration can be variable during different stages of injury, further altering the excretion of both nitrate and creatinine [18]. Previous studies [15, 16] have also shown substantial impact of diet on urinary nitrate concentration. Contrary to other studies investigating urinary nitrate concentration as a biomarker for disease e.g., Melichar et al. [19] researching inflammatory bowel disease, we did not restrict nitrate intake or exclude those not on a low nitrate diet. This was done to examine the use of urinary nitrate concentration in urgent clinical scenarios when a period of 48 h of a low nitrate diet would not be possible, such as here in kidney transplant rejection.

We used random spot urine samples which are familiar and acceptable to patients in the transplant setting. We have avoided the use of timed samples, which may offer a more accurate assessment [17] of uNCR, in order to be consistent with accurately assessing the use of uNCR as a biomarker in the clinically urgent setting of kidney transplant rejection.

We have shown that on an unrestricted diet and using random spot urines, urinary nitrate concentration remains hugely variable with no diagnostic threshold for rejection identifiable. Within patient values are also highly variable (Fig. 2) and unrelated to rejection status. Thus, it was not possible to produce individualised patient thresholds for rejection.

Our findings are notably different to the studies undertaken in this area by Smith et al. [4, 5]. In 1996 Smith et all [4] first reported that uNCR during renal transplant rejection was significantly higher than those with normal kidney function post transplantation (4937 μmole/g during rejection versus 1585 μmole/g during stable graft function). This was also found to be associated with elevated iNOS activity (6.4 pmol citrulline/min/mg protein compared with 0.51). Smith et al. [5] confirmed this in a second study in 2000 showing that urinary nitrate concentrations rose significantly from baseline to 4530 μM/litre during the − 5 to − 1 days leading to a formal diagnosis of renal transplant rejection. Interestingly Mugge et al. [8] reported a uNCR of 99 μmol/mmol in patients without cardiac transplant rejection and 131 μmol/mmol in patients with moderate/severe rejection however this difference was not significant. Mugge was only able to demonstrate a significant difference between patients with and without cardiac rejection when a small subgroup of 7 patients who had attended for repeated biopsies during the study time was analysed separately. In these patients it was found that the uNCR rose up to 99% from baseline and this value was higher, the greater the severity of rejection.

There are several potential reasons why these studies have different results to ours. Firstly, the severity of rejection is unclear from the two Smith papers. The Banff schema [3] used today was first published in 1991 and reviewed every two years thereafter. Prior to this there was no standardized, international classification for renal transplant biopsies which resulted in considerable heterogeneity. Smith et al. [4] published their first study in 1996 and do not explicitly state the criteria used for diagnosing rejection nor the grade or severity of rejection for comparison with our study. Thus, the patient cohorts studied by Smith and this paper, where levels of rejection are largely borderline, might have been substantially different.

Mugge et al. [8] showed, in a subgroup of patients who had repeated biopsies (7 patients, 48 biopsies), uNCR significantly rose by 99% during episodes of rejection with higher values for higher degrees of rejection. In our study we have 3 patients with repeated biopsies so cannot reliably assess this. From our data, we found no apparent indication that higher grades of rejection associate with increased uNCR (Table 4), though we acknowledge this is limited by low numbers. Similarly, we found no intra-patient thresholds for rejection.

Another potential difference between the present and earlier studies relates to drug effects on urinary nitrate. In this study 85% of our patient group are taking Tacrolimus with or without mycophenolate mofetil (MMF). It has been shown in animal studies that tacrolimus itself can cause a reduction in NO by reduction in endothelial nitric oxide synthase (eNOS) [12] as well as iNOS. MMF has been shown to have a similar effect [13]. Such a reduction in NO synthesis would be expected to reduce urinary nitrate. Given that tacrolimus could reduce iNOS expression, the enzyme used to generate NO during inflammation, it is possible that tacrolimus treatment contributes to that lack of rise in urinary nitrate in transplant rejection. Although the immunosuppression regime is not stated within Smith et al’s study [4], tacrolimus was only approved by the US Food and Drug Administration for prevention of renal transplant rejection in 1997 [20], a year after Smith et al’s [4] original study was published. In Smith et al’s [5] second study the use of ciclosporin is mentioned but not tacrolimus. The difference in the use of tacrolimus in the present and previous studies may contribute to the urinary nitrate concentrations being lower in our patients.

There are other important differences between our study and previous works. In the first Smith et al. [4] study, all of the patients were hospitalised and in the second [5] all samples were given within 90 days of transplantation. These circumstances are different to that of the well outpatient setting used in our study and may be associated with states of increased inflammation and increased iNOS expression.

Finally, at 55 years the mean age of our patient group is older than both Smith et al. [4, 5] and Mugge et al’s [8] studies (45, 45, 49 respectively). Given the age difference it is more likely that this is a more co-morbid population and represents the more liberal approach to transplantation in the U.K. now compared with the 1990’s. Disease states such as hypertension and cardiovascular disease as well as inflammatory conditions could all be affecting iNOS expression and nitrate concentrations. In addition, it has previously been shown that eNOS diminishes with age [21, 22]. It is not clear whether this would be clinically relevant in inflammatory states such as transplant rejection.

Our data have to be considered in context of its limitations. The number with biopsy proven rejection is small at 15. Furthermore, the most common Banff rejection grade diagnosed is borderline (7 cases) with few displaying more advanced stages of rejection (Table 4). The earliest that a patient was recruited post transplantation was day 6 and therefore we have no data from D0–5 post renal transplantation.

In summary, we have measured urinary nitrate concentration in renal transplant recipients using a method which could be applied to the clinically urgent setting of transplant rejection. In contrast to historical studies we have shown urinary nitrate concentration is not a useful biomarker for renal transplant rejection.

## Conclusion

Measurement of urinary nitrate concentration does not assist in the diagnosis of renal transplant rejection. Research should continue to focus on other more promising biomarkers to support the decision-making process around the earlier diagnosis of kidney transplant rejection.

## Data Availability

The datasets generated and analysed during the current study are available to access in the Royal Devon and Exeter Tissue Bank (RDETB) repository, (https://exetercrfnihr.org/about/rde-tissue-bank/) through application to, and approval from, the RDETB Steering Committee (see website for details).

## Abbreviations

uNCR: Urinary nitrate: creatinine ratio
iNOS: Inducible nitric oxide synthase
NO: Nitric oxide
